# Constricted Canals: A New Strategy to Overcome This Challenge

**DOI:** 10.1155/2014/564106

**Published:** 2014-05-11

**Authors:** Ricardo Machado, Emmanuel João Nogueira Leal Silva, Luiz Pascoal Vansan

**Affiliations:** ^1^Ribeirão Preto Dental School, University of São Paulo, 05508-070 Ribeirão Preto, SP, Brazil; ^2^Grande Rio University, 25071-202 Rio de Janeiro, RJ, Brazil

## Abstract

Negotiation of constricted canals can be a challenge during endodontic treatment. Over the years, several strategies have been presented in order to overcome the difficulties imposed by this anatomical feature. This paper presents three cases using a different protocol from that recommended by the manufacturer of the Protaper System in order to facilitate the negotiation of constricted canals. These cases suggest that the modified protocol shown is able to perform the shaping process with less resistance, reducing the risk of instrument separation and performing an effective process to reach the apical thirds in constricted canals.

## 1. Introduction

Negotiation of constricted canals is a well-reported challenge in endodontics [1, 2]. Over the years, several strategies have been presented in order to overcome the difficulties imposed by this anatomical feature. Some examples are the use of multiple radiographs as well as making sufficient access openings for proper visualization [3] and coronal preflaring in conjunction with copious irrigation and canal lubrication [2, 4]. Small dimensions files are required for pathfinding; however, these files should possess mechanical resistance to torsion and buckling so as to endure the loads imposed on them during apical progression [2]. Tapered files would have the rigidity but are too bulky to slide through a constricted space. Several manufacturers have attempted to manage this problem by altering tip and taper configuration in order to maximize the necessary balance between small size, increased rigidity, and minimal deformation [5, 6].

The Protaper System was developed by Dentsply Maillefer (Ballaigues, Switzerland) and features nickel-titanium instruments with a convex triangular cross section and progressively tapered design. These characteristics enhance the cutting action while decreasing the rotational friction between the file blade and dentin, minimizing instrument fatigue [7]. Protaper System has been evaluated by several methodologies measuring volume, surface area, thickness (diameter), and prepared surface, showing better results in comparison to other systems [8, 9]. However, the technique proposed by the manufacturer requires that the first finishing file (F1) reaches integrally the working length, hindering its use in constricted canals.

The aim of this paper is to show a report of three cases using a different protocol from that recommended by the manufacturer of the Protaper System in order to facilitate the negotiation of constricted canals.

## 2. Case Reports

### 2.1. Case 1

**Figure 1:**
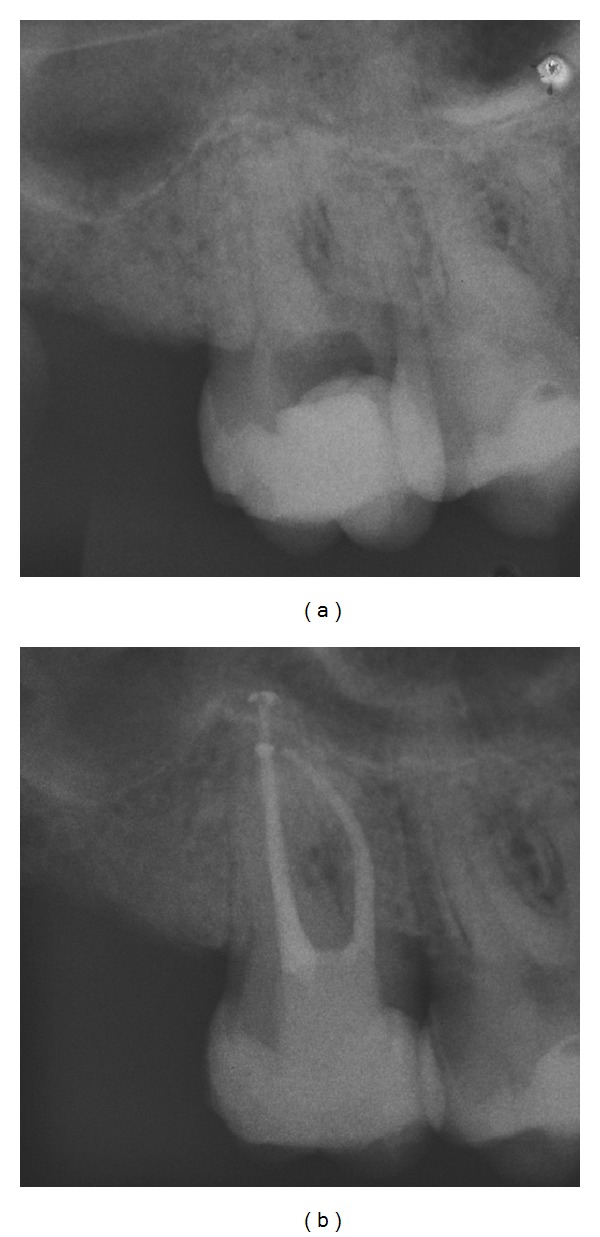
(a) Initial radiograph showing constricted canals mainly in the buccal roots. (b) Final radiograph.

A 54-year-old male with no general health problems was referred by his general dentist for the endodontic treatment of his right maxillary third molar. The tooth was extremely sensitive to cold stimulation and the percussion test was negative. Radiographic examination revealed the presence of constricted canals and no periradicular lesion (Figure 1(a)). Periodontal findings were within normal limits.

**Figure 2:**
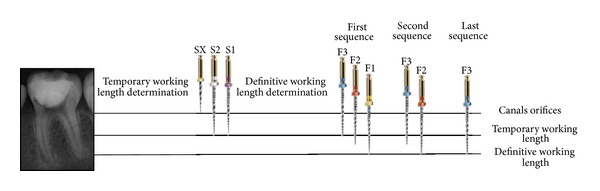
Sequence showing a different protocol for using Protaper System.

After the administration of local anesthesia with 4% articaine with 1 : 100.000 epinephrine (Articaine—DFL, Rio de Janeiro, Brazil), a rubber dam was placed and temporary restoration removal was performed in high speed rotation with diamond burs. Three canals were found and coronal flaring was achieved by using the Protaper SX, S1, and S2 rotary files (Dentsply Maillefer, Ballaigues, Switzerland) until the provisional working length (5 mm below the apparent length of the tooth). Definitive working length was verified by using the Elements Diagnostic Apex Locator (SybronEndo, Orange, CA, USA). Then, a crown-down instrumentation technique was performed using the following sequence (Figure 2):

- Protaper S2 (until resistance) and S1 (until the working length),
- Protaper S2 (until the working length),
- Protaper F3 (until resistance), F2 (until resistance), and F1 (until the working length),
- Protaper F3 (until resistance) and F2 (until the working length),
- Protaper F3 (until the working length).

1.0% NaOCl was used to irrigate the canals between each file. Patency files (10 K-File) were used between each file to prevent canal blocking. Then, the canals were flooded with 17% EDTA solution (Fórmula & Ação, São Paulo, Brazil) for 3 minutes and flushed with 0.9% saline solution. The canals were dried with sterile paper points and the obturation was performed with gutta-percha and epoxy resin sealer (AH Plus, Dentsply Maillefer) by the Tagger Hybrid Technique. The tooth received a temporary restoration (Cavit; ESPE, Seefeld, Germany) and the patient was referred back to the referring dentist for the definitive restoration (Figure 1(b)).

### 2.2. Case 2

**Figure 3:**
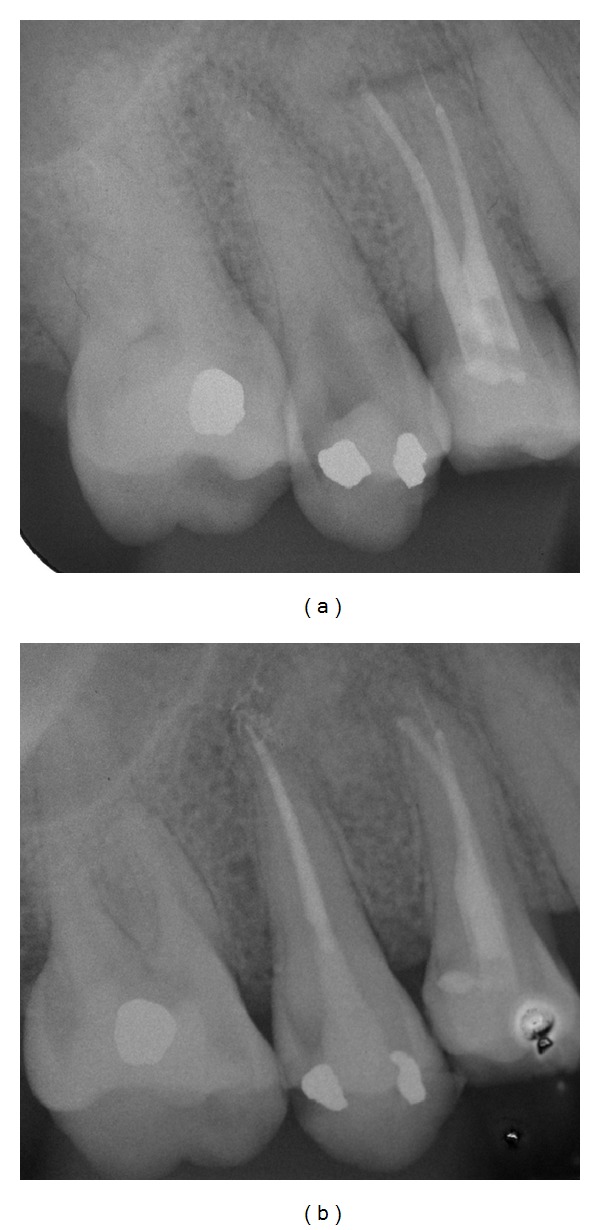
(a) Initial radiograph showing constricted canals. (b) Final radiograph.

A 34-year-old female with no general health problems was referred by her general dentist for the endodontic treatment of her right second maxillary premolar. The patient was submitted to an emergency visit two weeks before. The tooth presented expontaneous pain, which was increasing with vertical forces. Sensitivity test was negative. Radiographic examination revealed the presence of constricted canals and a small periradicular lesion (Figure 3(a)). Periodontal findings were within normal limits.

After the administration of local anesthesia with 4% articaine with 1 : 100.000 epinephrine (Articaine—DFL, Rio de Janeiro, Brazil), a rubber dam was placed and temporary restoration removal was performed in high speed rotation with diamond burs. Two canals were found and the same technique demonstrated in case 1 was performed.

After instrumentation and obturation, the tooth was restored with Cavit (ESPE, Seefeld Oberb, Germany) and the patient was referred back to the referring dentist for the definitive restoration (Figure 3(b)).

### 2.3. Case 3

**Figure 4:**
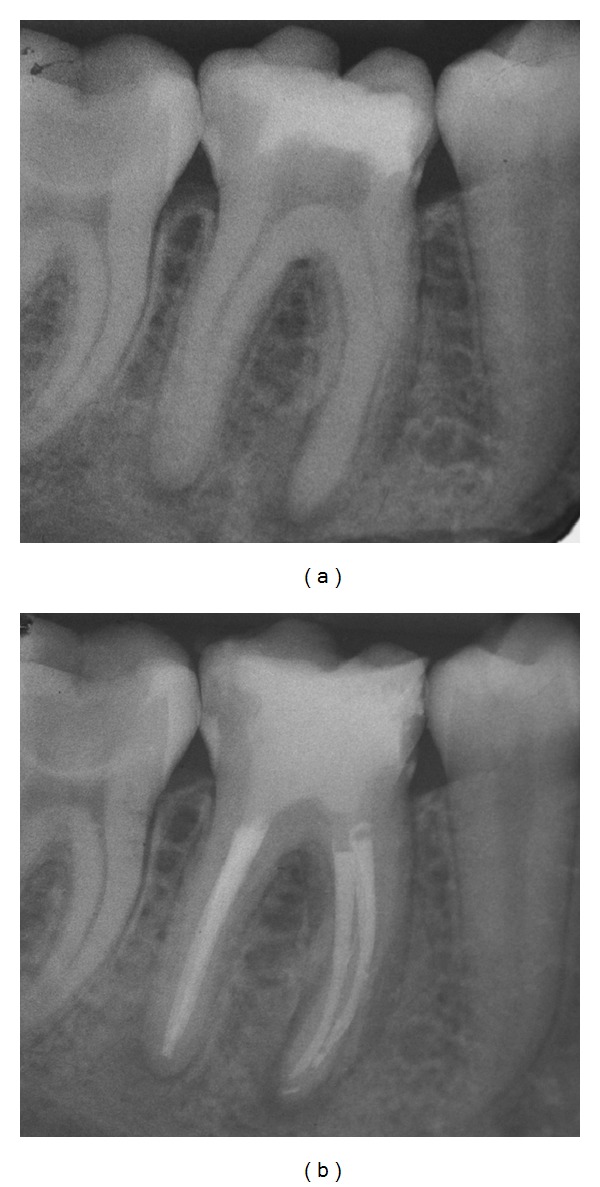
(a) Initial radiograph showing constricted canals mainly in the apical and medium thirds of the mesial root. (b) Final radiograph.

A 39-year-old female with no general health problems was referred by her general dentist for the endodontic treatment of her right first mandibular molar. The patient was submitted to an emergency visit two weeks before the appointment. The tooth presented spontaneous pain that increased with vertical forces. Radiographic examination revealed the presence of constricted mesial canals and a periradicular lesion in both roots (Figure 4(a)). Periodontal findings were within normal limits and the medical history was noncontributory.

After the administration of local anesthesia with 4% articaine with 1 : 100.000 epinephrine (Articaine—DFL, Rio de Janeiro, Brazil), a rubber dam was placed and temporary restoration removal was performed in high speed rotation with diamond burs. Three canals were found and the same technique demonstrated in case 1 was performed. After instrumentation and obturation, the tooth was restored with Cavit (ESPE, Seefeld, Germany) and the patient was referred back to the referring dentist for the definitive restoration (Figure 4(b)).

## 3. Discussion

Reaching the apical part of the canal is usually a challenge for the endodontist. The reason for this may be a constriction of the root canal [10] or severe curvature of the root canal [11]. Regardless of whether operators can recognize the reason for inaccessibility, they must take proper strategies for negotiation. If the canal is not constricted but is severely curved near the apical foramen, which prevents negotiation, operators may try to negotiate the canal with a precurved file. However, if the canal is totally constricted near the apical foramen, accidents like ledges, perforations, or transportations can occur during the exploration [12].

In cases of constricted canals, an excessive pressure applied on the handpiece [13], associated with the contact area between the canal walls and the cutting edge of the instrument [8] and/or when the canal cross section is smaller than the nonactive or noncutting tip of the instrument, can cause what has been described as taper lock, a phenomenon usually occurring with regularly tapered instruments [14]. This risk may be reduced by performing coronal enlargement and by creating a manual and/or mechanical glide path before using NiTi rotary instrumentation [8, 15, 16]. Following this initial shaping, using the Protaper System in a different protocol from that recommended by the manufacturer was able to promote a better overall result in cases of constricted canals. Two effective advantages can be stated when using the instrument in a crown-down manner. First, two initial thirds of the canal are shaped by the strongest portions of the files. In this way, a coronal flaring is done more effectively than when using the conventional protocol. Therefore, using that system in the previously described manner, the tips of the files are free of great dentin stresses, considerably reducing the chances of fractures.

Our clinical observations suggest that the modified protocol shown is able to perform the shaping process with less resistance, reducing the chance of instrument separation and performing the most effective process to reach the apical thirds in constricted canals. This observation is interesting because it is possible to feel that, initially, the most effective contact between the great taper file portions and the dentin walls of the initial thirds would provide dissipating excess forces doing a linear shape and an easier access to deeper portions of the canals.

## 4. Conclusions

The authors' clinical experience points to the feasibility of this technique in cases of constricted canals; however, the authors believe that this should be tested by different clinical and research work in order to enable its safe use and effective and scientific support.
